# Towards practice change: a qualitative study examining the impact of a Child Psychiatric Access Program (Project TEACH) on Primary Care Provider practices in New York State during pandemic times

**DOI:** 10.1186/s12913-023-09999-z

**Published:** 2023-09-13

**Authors:** Nayla M. Khoury, Alex Cogswell, Melissa Arthur, Maureen Ryan, Eric MacMaster, David Kaye

**Affiliations:** 1 Norton College of Medicine at SUNY Upstate Medical University, Syracuse, NY 13210 USA; 2 Jacobs School of Medicine and Biomedical Sciences, University at Buffalo SUNY, Buffalo, USA; 3 Family Medicine Residency Program, St. Joseph’s Hospital Health Center, Trinity Health, Syracuse, USA

**Keywords:** Integrative care, Child Psychiatric Access Program, COVID19, Mental health crisis, Practice transformation

## Abstract

**Background:**

This study aims to explore the perceived impact of Project TEACH (Training and Education for the Advancement of Children’s Health), a New York State Office of Mental Health funded Child Psychiatric Access Program (CPAP), on pediatric Primary Care Providers (PCPs) and their practice. Practice change over time was assessed in the context of rising mental health needs and in the context of COVID19 pandemic.

**Methods:**

Focus groups utilizing a semi-structured format were conducted with pediatric PCPs who have been high utilizers of Project TEACH over the past 5–10 years and PCPs in similar regions who have been low or non-utilizers of the program. The semi-structured interview focused on practice change, asking about pediatric mental health, practice setting and flow, professional development, and changes over time in the context of COVID-19 pandemic and Project TEACH.

**Results:**

Themes identified include increasing confidence of PCPs, particularly those who are high utilizers of the phone consultation line, increased routine use of screening and comfort bridging pediatric patients with mental health needs. Challenges include rising mental health needs, inadequate mental health services, difficulties with family follow through and high emotional burden on PCPs caring for these patients. In this context, participants noted that collaboration with Project TEACH provided needed emotional support.

**Conclusions:**

Integrated care and CPAPs such as Project TEACH are vital to helping PCPs handle rising mental health needs particularly in current crisis times. Ongoing systemic challenges accessing care remain and contribute to emotional burden placed on pediatric PCPs.

**Supplementary Information:**

The online version contains supplementary material available at 10.1186/s12913-023-09999-z.

## Background

Children’s mental health has been a leading public health concern for at least the last two decades [1]. During the current COVID19 pandemic, this has only intensified. While 13–20% of children and adolescents have current psychiatric disorders, many, if not most, children still receive little or no services [2, 3]. The point prevalence estimates in the first year of COVID19 pandemic were reported even higher; a recent global meta-analysis from 2020 noted depression estimates of 25% and anxiety estimates of 20% [4]. Families facing structural racism and families experiencing socioeconomic disadvantages have been disproportionately affected both by the COVID19 crisis and gaps in mental health care [5, 6]. In this context, the American Academy of Pediatrics (AAP), the American Academy of Child and Adolescent Psychiatry (AACAP) and Children’s Hospital Administration (CHA) declared a national emergency in child and adolescent mental health.

One strategy to address this public health crisis is to target primary care clinicians (PCPs) to become more skilled and confident in meeting the pediatric public mental health need. An important strategy to accomplish this is integrated care, which includes a spectrum of approaches including behavioral health embedded within primary care, team-based collaborative care models, and Child Psychiatric Access Programs (CPAPs). CPAPs have become established widely in the United States with 47 states now sponsoring programs [7]. CPAPs were developed originally to support efficient diagnosis and treatment of mild to moderate mental health issues within primary care with the support of statewide child psychiatry phone consultations, face-to-face consultations, and care coordination. These programs have become increasingly well-established and utilized, yet most existing literature is descriptive in nature [8]. A recent meta-analysis of randomized controlled trials (RCTs) with behavioral health integration in pediatric primary care found significant effects for integrated care versus usual care conditions (d = 0.32; 95% CI, 0.21–0.44; *P* < 0.001). The strongest effects were noted for integrated care models that include collaborative care or emphasize behavioral health practitioners and PCPs working together typically with a care manager to improve health and behavioral health [9]. In addition to enhancing patient outcomes, these programs have been shown to lead to increased confidence of PCPs in addressing mental health concerns in the primary care setting [10].

Project TEACH is a New York State Office of Mental Health funded CPAP that has provided consultative services and training to PCPs in New York State for over a decade. Project TEACH was established in 2010 as a coordinated care program in New York as a consortium of 5 academic centers to provides real time phone consultation, rapid face to face consultations, assistance with linkage/referral, and formal education programs for pediatric PCPs across the state [11]. PCPs also receive education regarding the spectrum of behavioral health resources in their area and ways of increasing access, along with patient-specific linkage/referral information. Families who have received face to face consultations are directly assisted in identifying helpful resources and navigating care systems. In 2019, perinatal psychiatric consultation support was added as a second focus of the program. Project TEACH services are currently provided through a consortium of 7 academic psychiatry departments across New York consisting of 15 child and adolescent psychiatrists, 10 reproductive psychiatrists, 2 maternal mental health psychologists, and 6 Liaison Coordinators. There is one toll free phone number to access all services. To date, Project TEACH has enrolled 5,731pediatic primary care clinicians, and completed 23,517 child psychiatric phone consultations and 3227 face-to-face evaluations of children and adolescents. Over 1500 pediatric PCCs have completed the flagship educational program; The REACH PPP program in collaboration with the REACH institute was utilized for the first 6 years in partnership with Project TACH faculty; subsequently Project TEACH developed its own Intensive Training program for the last 6 years. In 2022 two-week follow-up surveys following consultations confirmed that 97% of consultations were very helpful or extremely helpful. 100% would recommend the program to other PCPs. These percentages have been consistent across all years of the program. Gadomski conducted a qualitative evaluation of Project TEACH using a semi-structured interview of 40 pediatric PCPs and demonstrated that PCPs who were trained through Project TEACH report more confidence interacting with families about mental health, assessing severity, and providing treatment, including prescribing psychotropic medications [12]. Kerker conducted a quantitative analysis of the New York State Medicaid database and found an increase in recognition and prescribing in those PCPs trained by Project TEACH [13]. Foy and colleagues have also highlighted the broadening scope and increasing complexity of mental health challenges in practices of PCPs over time [14]. While the current data supports the perceived benefit of programs such as Project TEACH, less is known about the effect of collaborative care models on practice change and the experiences of primary care providers to meet the rising mental health needs of their patients, particularly in the context of the current COVID19 pandemic. Thus the aims of this study were 1. To explore the experiences of primary care providers in New York State with practice change, specifically understanding ways they and their practices may have shifted to meet the needs of pediatric mental health cases over time and in the context of COVID19 pandemic and 2. To understand the potential perceived impact of Project TEACH on practice change.

## Methods

This study examined the experience of PCPs handling pediatric mental health issues and explored the perceived impact of a collaborative care program and other factors on practice change. Focus groups were conducted in 2021 with PCPs who have been high utilizers of Project TEACH over the past 5–10 years and PCPs in similar regions who have been low or non-utilizers of the program.

### Targeted sample

Participants included PCPs in areas of New York covered by Project Teach and who met one of three general utilization categories:

- High utilizers, defined as top 10 callers annually in each region, and/or calling 7 or more times annually. A subgroup of high utilizers was a group of four PCP champs, who, in addition to high utilization of Project TEACH’s phone line, had qualified to be invited to become trainers with the REACH Institute.
- Low utilizers defined as PCPs who have utilized the phone consultation line 3–7 total times.
- Non utilizers defined as non-registered or registered recently (within the last month or who have called the phone consultation line less than 3 times).

### Recruitment

PCPs that utilize Project TEACH and were classified as either high or low utilizers received a recruitment email. Non utilizers were recruited from those PCPs who signed up for Project TEACH’s statewide virtual intensive training, as this is how many PCPs are introduced to Project TEACH. All participants had therefore signed up for educational training with Project TEACH. The recruitment email invited PCPs to participate in a focus group, and included the purpose of the project, stated that all participation is voluntary, and has no bearing on participants’ ability to continue to utilize services.

### Focus group procedures

Focus groups were conducted virtually over Zoom, and began by reviewing informed consent for the study. Focus groups were one hour each with two facilitators (NK, SS, MR, MA, EM). A semi-structured interview tool was used to focus the discussion on questions related to the following themes: 1. Pediatric mental health in PCP practices 2. Practice changes over time 3. Professional development 4. Practice flow 5. Impact of Project TEACH and 6. Impact of COVID19 pandemic (see Appendix for interview questions). The content of the group was recorded on zoom, and subsequently transcribed.

### Data analysis

After de-identifying participant data, themes were coded using an iterative process of creating a codebook from the data using grounded theory principles with three coders (AC, MA, SS). This analysis was completed in a series of steps. First, all coders independently reviewed participant data and jointly generated a tentative codebook, with both primary and secondary codes identified. Second, two coders (AC & MA) then independently assigned primary and secondary codes to excerpted segments of focus group content. A third coder (NK) then reviewed the two sets of codes and calculated rate of convergence within each primary and secondary category. Given an overall convergence rate of 49%, a meeting was held with AC, MA and a third coder (NK) to discuss, refine codes, and resolve discrepancies. The consensus coding achieved is what is presented in this manuscript. IRB from SUNY Upstate reviewed the study and determined it to be waived from review given that no identifiable personal information was used for the purpose of this study.

## Results

A total of 22 PCPs completed one of the 8 focus group interviews. Table 1 shows the participants by non-identifying demographics and degree of Project TEACH utilization. A total of eight focus groups were held with varying numbers of participants. One focus group of nine high utilizers was unable to be transcribed and coded due to a recording error and is not included in the results. High utilizers and PCP Champs were older than non or low utilizers (average age 56 for PCP champs and 58.9 for high utilizers v 45.5 for low or non-utilizers) and had been in practice longer (29 years on average vs 12 years for low utilizers and 14.6 for non-utilizers). High utilizers practiced in a range of settings whereas low utilizers all practiced in rural settings and non-utilizers in urban settings. Due to small sample size and no meaningful differences in response, analysis of data combined the low and non-utilizers into one group and the high utilizers (including PCP Champs) into another group.

**Table 1:** Participants and Project TEACH participation

	Non Utilizers	Low Utilizers	High Utilizers	PCP Champs
Number of Participants	*N* = 5	*N* = 3	*N* = 10	*N* = 4
Gender	Female = 4 Male = 1	Female = 2 Male = 1	Female = 7 Male = 3	Female = 3 Male = 1
Age	Average Age = 45.5 Range: 32–56	Average Age = 45.5 Range: 42–49	Average Age = 58.9 Range: 46–73	Average Age = 56 Range: 45–69
Specialty	Pediatrics = 4 Family Medicine = 1	Pediatrics = 1 Family Medicine = 2	Pediatrics = 9 Family Medicine = 1	Pediatrics = 3 Family Medicine = 1
Practice Location	Upstate = 0 Downstate = 5	Upstate = 3 Downstate = 0	Upstate = 7 Downstate = 3	Upstate = 2 Downstate = 2
Practice Setting	Urban = 5 Urban/Suburban = 0 Rural = 0	Urban = 0 Urban/Suburban = 0 Rural = 3	Urban = 1 Urban/Suburban = 4 Rural = 4	Urban = 2 Urban/Suburban = 1 Rural = 1
Years in Practice	Average Years = 14.6 Range: 2–25	Average Years = 12 Range: 9–17	Average Years = 29.6 Range: 17–39	Average Years = 29 Range: 19–41
Years Utilized Project TEACH	Average Years = 0 Range: N/A	Average Years = 7.7 Range: 7–9	Average Years = 9.6 Range: 6–12	Average Years = 11.25 Range: 11–12

### Themes

**Fig. 1:**
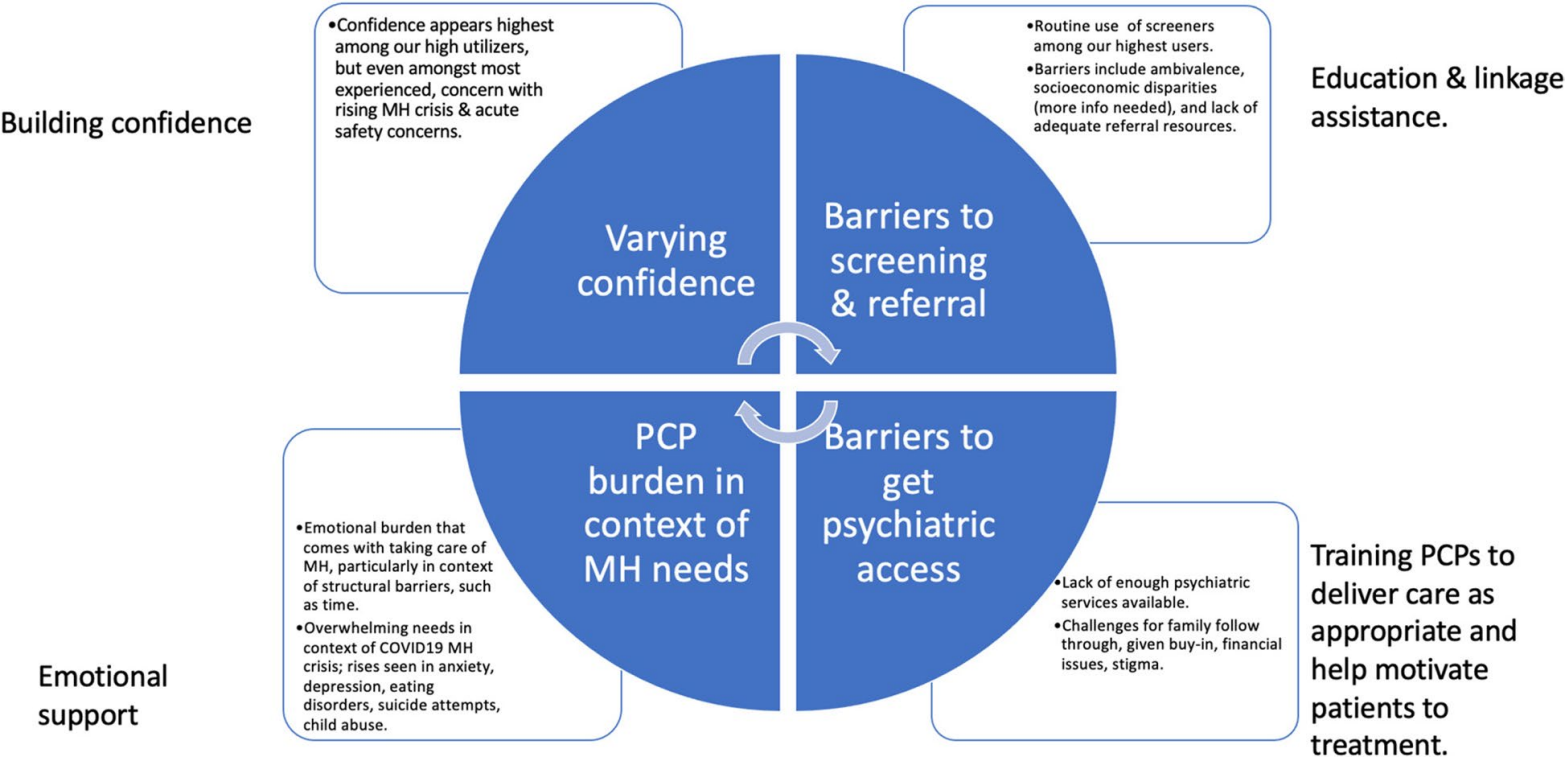
Towards practice changes: Project TEACH’s impact on current practices & ongoing challenges

Blinded coders reviewed the transcripts and identified several themes across the interviews. Table 2 highlights themes and subthemes identified with illustrative quotes. Responses are categorized as related to positive changes (e/g/ confidence and clinical skills, systematic approaches to care, and emotional support) and challenges (e.g. access issues. SDOH, family stress and reluctance, PCP emotional toll) (see Fig. 1). PCPs described various factors involved in practice transformation due to utilization of Project TEACH services, including increased confidence in assessing and treating mild to moderate psychiatric disorders, leading to increased utilization of routine screening and referrals, and improved ability to identify those who need specialized psychiatric care, with skills to motivate patients towards treatment. As Fig. 1 depicts, all PCPs named ongoing challenges to their work, particularly in the context of COVID19 pandemic.

**Table 2:** Themes and illustrative quotes

Themes	Practice changes	Project TEACH benefits	COVID19 impacts	Barriers to MH resources	PCP challenges & burdens
**Subthemes** “illustrative quote(s)”	Routine screening & referral “…we definitely have increased our screening forms.”	Continuity “We have talked a bunch about a few patients over years.” “Over the years, having gotten to know the psychiatrist that I’m talking to, who I know and who knows me	Social isolation “People feel really isolated and I think kids… feel defeated especially with COVID, like they can’t be with their friends, they can’t do school effectively.”	Financial & insurance issues “It’s not easy to get our patients to see anyone to help them whether it’s a psychiatrist, psychologist, social worker, almost everybody doesn’t accept insurance.”	Time constraint “My time is a barrier.” “It’s not just the visit; it’s the visit and the after visit and calling the parents and calling the therapist, speaking to the nutritionist, and trying to coordinate care and then following gup and then dealing with acute crisis because they are not doing well. You know, it’s exhausting.”
	Telemed. utilization during pandemic “a telemed visit allows me to actually see them when their parents might not have brought them in person” “We have implemented telehealth but its been challenging…” “I don’t feel trained in that”	Emotional support “Project TEACH is to some extent helping take care of those people who take care of children.” “To feel supported is just huge cause it makes me feel like okay I can do this stuff if I know I have backup.”	Mental health crisis for patients & families “about 60 percent of my patient population is now anxiety, depression & eating disorders…and its unbelievable just how much need and how much the kids and families are in crisis.”	Lack of access “They screen positive and then its virtually impossible to get them plugged in for mental health treatment.” “It’s astonishing to me how little services really exist.” “The child is in crisis, the only resort I have is to send them to the ER…”	Emotional burden “I am personally getting burned out… I’m the only one in my practice that prescribes any medications.” “It’s landing in my lap and my heart breaks every single time.”
	Co-located or integrated care “The thing that really helped us the most for two years, we had a psychologist that was co-located with us.”	Face to face feedback “I was really impressed with the depth of the evaluation.”	Pandemic specific difficulties “Families that had difficulty keeping structure in the house and kids with ADHD who really need structure.”	Family buy-in issues “Sometimes it’s the barrier of their parents.” “They don’t feel like [mental health] is a problem and they are not interested in medication definitely and may be convinced to do counseling.”	Just one consult and then what? “I can get one consultation. Often it’s not enough.”
		Diagnostic clarification “Reassess and make sure I’m on the right track…”	Social unrest “I’ve had many parents burst into tears in the office. Like how terrified they are for their young black and brown boys. I guess I hadn’t realized the level of anxiety that parents hold even with young kids.”		
		Improved confidence “Before Project TEACH, I didn’t ask because I didn’t want to know because I had no way to manage them. I feel much more comfortable starting an SSRI. I feel like I can give the teens the tools for how to manage anxiety. I can do some motivational talking in terms of setting goals for the depressed teen while they wait to get into therapy.”

#### Positive changes: Improved confidence and skills

Indeed, across the varying levels of Project TEACH utilization, participants highlighted how Project TEACH improved their confidence in assessing and treating pediatric mental health cases. Confidence is inextricably related to improved skill and knowledge. A participant in the high utilization group noted,

Before I did Project TEACH, I didn't ask because I didn't want to know because I had no way to manage them. I feel much more comfortable starting an SSRI. I feel like I can give the teens some tools for how to manage anxiety. I can do some motivational talking in terms of setting goals for the depressed teen while they wait to get into therapy

Among those who have had the highest level of utilization and training, it was noted that follow-up calls with the consulting Child and Adolescent Psychiatrists after first phone consultation helped to increase comfort in prescribing new medication. Specifically, discussing a consultant’s rationale for making specific medication choices during follow-up helped PCPs generalize knowledge and boost confidence for the next time they may be faced with a similar complaint. Said one high utilizer,

I can’t even begin to say how much a single call may do. I would suggest that anytime you have a new condition which you haven’t spoken with Project TEACH, whether its ADHD, depression, anxiety and you’re deciding what to do, call in…do a telephone consultation. And do it when you can speak for 15 minutes because you’ll hear so much about it plus the reasoning of doing things a certain way.

#### Positive changes: Screening and more systematic approaches to care

PCP champs who have utilized Project TEACH the most, including phone consultation and participating in educational training, described their increased comfort with asking about psychiatric illness leading to more systematic approaches to care, including screening, utilization of rating scales, and population health approaches. As one participant identified,

[in the past], it was definitely not done very well, largely because opening Pandora’s box led to a place where I didn’t really know what to do. And so I think that our screening has gotten better and better as we’ve had a better management system.

Systematic approaches included general screening at well visits, but also investigating potential underlying psychiatric illness when patients presented with common complaints. Reported one PCP champ, “I have much more confidence in assessing for anxiety early on versus doing every test known to mankind and sending them to GI and waiting for the stool studies.” This change was noted to reduce the need for over-referral or over-utilizing costly diagnostic tests.

Practice transformation for some PCPs entailed no longer relying on Project TEACH as often for simpler cases; those who were previous high utilizers of Project Teach but now low utilizers noted that their confidence and comfort may be one reason they reach out less. One participant noted, “The help with like clarifying a diagnosis is huge. I feel like one of the reasons I haven’t called as much as I’ve gotten a little better at it.”

#### Challenges to providing mental health care in the primary care setting: Access to mental health providers worsened by COVID-19 Pandemic

No matter the degree of utilization, PCPs described challenges with accessing appropriate mental health services. Even within clinics with integrated care that relied less on Project TEACH, PCPs highlighted the immense challenges involved in accessing mental health care, reporting that mental health providers often were overwhelmed with patient load with saturated capacity. PCPs described more burden because of not having needed mental health support. These challenges were highlighted as particularly relevant in the context of the COVID19 pandemic and the current mental health crisis. Themes elicited among participants included the continual and expected rises in anxiety, depression, eating disorders, suicide attempts, and child abuse. Stated one PCP champ:

I would say about 60 percent of my patient population now is anxiety and depression and eating disorders. And it’s unbelievable the just how much need and how much the kids in the families are in crisis.

In this context, many PCPs described additional pandemic-specific difficulties getting their pediatric patients needed mental health services. One such difficulty was understandable hesitancy to get any services in the early stages of COVID19 pandemic. One participant reported, “The children are not going into the clinics. But definitely there is hesitancy to even go into any facility.” Another PCP commented:

I’m already schedule busted before I even start asking questions about it and then it becomes even more of a schedule buster, then there’s no one to refer them to. Like we have like literally one health service in the area that we’ve gotten like three kids into a social worker. But when I ask someone, “Do you guys have more resources?”, they’re like, “not really.” It’s really hard to get into anywhere. I think it’s a systemic level issue of connecting.

#### Challenges: Social determinants of health (SDOH) and increased family stress.

It is well documented that SDOH can limit access to quality education and health care resulting in poor health and education outcomes [15] Families and caregivers experiencing barriers to care or treatment or who have been disadvantaged by systemic policies of exclusion such as structural racism can struggle with navigating the complexity of the health care system [16]. As a result, follow up and follow through on PCP recommendations may be obstacles for these pediatric patients, even after mental health concerns have been identified. One participant noted,

And then you know some of them will show up and then they would discover they haven’t been in school since the pandemic. And then try to dig into why. And it’s been the iPad that they couldn’t figure out how they work because the mother is illiterate…This child is not getting any services. They are not going to school. They are not going to any appointments. And I know it’s simply a social economic disparity issue. It’s not that the mother doesn’t care or is purposely neglecting their child. They just can’t navigate the system…

#### Challenges: Parent/family reluctance

Another related barrier to accessing care is family reluctance, described among low and high utilizers alike. Reluctance might be due to stigma or not believing in mental health or mental health treatment or be related to denial, and becomes an added barrier to connecting youth to care. A participant noted that many families they serve do.

Not want to be involved in mental health at all. The don’t feel like it’s a problem and they are not interested in medication definitely. They may be convinced to do counseling…

Stigma surrounding mental health services becomes compounded by scarcity of resources, financial barriers and PCP time constraints. As one participant noted:

Basically, like everywhere else, even all the mental health centers in New York are really not accepting new patients. And then the buy-in thing double bites that because the families don’t go to their appointments…

#### Challenges: PCP Emotional Toll

In this context, PCPs note having insufficient resources for patients who need help. This can lead to a sense of moral injury or burnout. As one participant noted, “As effective as I feel, I feel really defeated because no matter what I do, the floodgates are opening up.” The emotional burden is exacerbated by the limitations of time in primary care. Stated one participant,

I think the issue is almost more that this takes a lot of time and it's not like a 10 or 15 minute break where you're in and out and this is what you have to do. You have to invest a lot of time. It's emotionally draining. And I think some people are just saying, ‘I don't want to spend the time; this is not what I went into.’

In the face of these challenges, participants noted the emotional support that Project Teach provided the PCPs themselves. One participant noted, “Project Teach is to some extent helping take care of those people who take care of children.”

#### Differences between high and low/non utilizers

PCPs who were high utilizers of Project TEACH described transformation of their practice through increased systematic screening and feeling more comfortable being able to assess, triage, and treat selected pediatric mental health complaints. They noted increased confidence using techniques to motivate their patients to engage with mental health providers when needed and to begin treatment in the interim.

PCPs who were low utilizers expressed ambivalence about their confidence in treating pediatric mental illness, noting that they had learned how to start a medication through recent training, but still felt uncomfortable managing patients with psychiatric conditions. One such PCP noted,

I never used to prescribe any antidepressant or anything because I just didn’t feel comfortable. Like I didn’t know what doses to start at so at least with that first training... I took pictures of the slides so I would know what dose to start at. And so, you know I feel comfortable starting it; I just don’t think I have the expertise to manage it.

All groups reported significant barriers to accessing mental health services for their patients, including scarcity of resources, socio-economic barriers, and stigma, or lack of buy-in*;* pediatric mental health needs intensified during COVID19 pandemic, as did the barriers to care. These themes highlight the importance of ongoing phone consultation and follow up calls in boosting PCP confidence and skills to tackle pediatric mental health complaints; for many, these calls also serve to support PCPs own sense of emotional burden.

## Discussion

Since the inception of Project TEACH, mental health needs of children and adolescents have been increasing in frequency and intensity and PCPs are often the frontline providers. When we look at the current landscape of mental health and reflect on the way it has changed since the beginning of the COVID19 pandemic, we are often struck with major themes such as increased symptom severity, increased mental health needs, lack of access to mental health care, and increased burden on disadvantaged families, many of which were highlighted in our discussions with primary care providers.

In this study we aimed to obtain qualitative data from PCPs who have utilized Project TEACH to understand practice change over time and compare to low or non-utilizers of the program. The aim of Project TEACH has been to “teach PCPs to fish,” to increase their ability to assess and manage mild-moderate mental health concerns within the pediatric primary care setting. The current study can better target future efforts at understanding the systemic impact of CPAPs on PCPs. The public health need for pediatric mental health services was high and largely unmet before COVID19 and in the past 2–3 years has only grown greater, making the importance of CPAPs even more crucial.

Given the complex situation currently, these focus groups were meant to better understand and expand upon ways that Project TEACH can be helpful in its current iteration, and to extrapolate where we may go in the future. As our results demonstrate, we have reasonable evidence to support that with higher frequency of PCPs utilizing services, there is a greater feeling of confidence in managing pediatric mental health challenges, which is supported by previous studies [12, 13]. This confidence appears to stem from greater knowledge and skills in assessing and managing mild-moderate mental health concerns. High utilizer PCPs endorse evidence-based approaches to assessment including regular use of rating scales and questionnaires. Further, they have seen the importance and impact of addressing mental health concerns in systematic ways within their practices. They appreciate that they cannot do this alone and this requires a team-based approach throughout the practice. Low utilizers were unlikely to be in practices that incorporated systematic practice change. They also were less likely to have the confidence to approach mental health concerns, making it less likely that the children in their practices will access mental health services if needed and may further add to stigma.

Reflecting upon these two metrics, we can see that with consultation support and education in following evidence-based interventions, primary care providers feel more competent and confident in screening/diagnosing common pediatric mental health concerns and subsequently initiating intervention and managing acute safety concerns. This paper adds to the growing literature that supports the further expansion of integrated care generally, and CPAPs specifically, to address the pediatric mental health crisis we are facing. As pediatric mental health needs grow and outpace the graduation of pediatric mental health providers (psychiatrist, psychologists, social workers, therapists), this role will increasingly be pushed to primary care, further underscoring the crucial need to prepare and support PCPs as they face this challenge.

### Limitations

While there are several strengths of this study (PCPs recruited throughout the state, with varying settings, practices, and levels of contact with Project TEACH), there are also two major limitations. The first is the small number of participants, which may limit generalizability. Given that recruitment for focus groups relied on some contact with Project TEACH, there may be selection bias in terms of eliciting experience of PCPs who may see the need for Project TEACH. PCP practices with more embedded behavioral health models of integrative care may have different experiences of practice transformation and challenges. However, our low utilizer group provided some insight into ongoing and similar challenges to mental health access issues even in these settings. A second major limitation is that the high utilizer group had been in practice double the time (29 years vs 14) which may confound results. Practice experience alone may contribute to increased confidence with assessing and treating pediatric mental health or contribute to greater knowledge of resources such as Project TEACH. An area for continued focus is targeting PCPs in-training and recent graduates to get trained earlier and become familiar with resources such as Project TEACH. Of note, another limitation to our study may be attributable to regional practices, especially important to note given the broad geography and population density/access to resources across New York state. Our results demonstrated a similar outcome to a 2021 study by Cotton et al. regarding accessing CPAPs in Maryland [17], namely that low utilizers tended to practice in rural areas whereas non-utilizers tended to be located in more urban settings. We have a limitation here that our results may indicate regional practice factors (rural PCPs likely to be managing mental health concerns given lack of resources, may have become more proficient and subsequently used the consultation less frequently whereas non-utilizers may be a result of greater service access in the urban areas they practiced in) more than a true reflection of PCP utilization. Lastly, the focus group facilitators were affiliated with Project TEACH, which certainly may have influenced responses. However, the executive director of Project TEACH did not participate in any focus group facilitation.

## Conclusions

CPAPs are increasingly utilized in the United States to try to address the mental health crisis in children and increase access to services by bolstering PCPs. In this qualitative study, we conducted focus groups with 22 PCPs with varying degrees of contact with a large CPAP, Project TEACH. Increased knowledge, skills, and confidence were evident for high utilizers. Practice change was also highlighted by the high utilizers. Low utilizers had less confidence and were less likely to approach mental health concerns in their primary care setting. An important area to consider is the impact of CPAPs on the emotional well-being of PCPs, which was often mentioned by high utilizers. This highlights new areas of focus for our program and CPAPs. Many challenges remain to get children and adolescents the mental health care they deserve. The UPSTF recommends all adolescents be screened for depression and anxiety, and primary care physicians play an important role in mental health care, With the support and consultation of CPAPs, more pediatric patients with mental health needs can receive access to the quality care they need.

### Supplementary Information

**Additional file 1: Appendix. **Semi structured interview tool.

## Data Availability

The datasets used and/or analyzed during the current study are available from the corresponding author on reasonable request.
