# Supplementary material for: Towards practice change: a qualitative study examining the impact of a Child Psychiatric Access Program (Project TEACH) on Primary Care Provider practices in New York State during pandemic times
Source: BMC Health Serv Res. 2023 Sep 13;23:985. doi: 10.1186/s12913-023-09999-z (PMC10500716; doi:10.1186/s12913-023-09999-z)
Supplement: Supplementary file 1 — Additional file 1: Appendix. Semi structured interview tool. [file 12913_2023_9999_MOESM1_ESM.docx]

**Appendix**

**Semi Structured Interview Tool**

- Pediatric mental health
  - What are the main mental health issues faced by your patients?
  - How prevalent are MH issues  in your practice?
  - Have you seen any changes in issues over time?
- Practice change
  - What is the most significant thing you have done in your practice to meet the MH needs of your patients?
  - What has been the greatest barrier in your practice to meet the MH needs of your patients?
- Professional development
  - What impact have Project TEACH phone consultations/face to face evaluations had on your comfort addressing mental health issues in your patients?
  - What impact have Project TEACH Core or Statewide trainings had on your comfort addressing mental health issues in your patients?
  - How has the complexity of your consultation questions changed over time?
  - How would you rate your comfort/proficiency with mental health before Project TEACH?
  - How would you rate your comfort/proficiency with mental health after Project TEACH?
- Practice setting/flow
  - What is the current workflow like in your office?
  - How has mental health management for your patients changed in the time of COVID?
  - What do you feel is going well?
  - What needs to be improved?
- Impact questions
  - How has Project TEACH or the CAP/LC relationships over time  impacted your practice?
  - What would be most helpful to increase your confidence dealing with mental health in primary care?
  - Would you recommend Project TEACH to your colleagues?
- COVID impacts
  - How has the COVID pandemic changed how you manage your patient’s mental health issues?
